# Kinetic Characterization of Exonuclease-Deficient *Staphylococcus aureus* PolC, a C-family Replicative DNA Polymerase

**DOI:** 10.1371/journal.pone.0063489

**Published:** 2013-05-16

**Authors:** Indrajit Lahiri, Purba Mukherjee, Janice D. Pata

**Affiliations:** 1 Wadsworth Center, New York State Department of Health, Albany, New York, United States of America; 2 Department of Biomedical Sciences, University at Albany School of Public Health, Albany, New York, United States of America; Institute of Molecular Genetics IMG-CNR, Italy

## Abstract

PolC is the C-family replicative polymerase in low G+C content Gram-positive bacteria. To date several structures of C-family polymerases have been reported, including a high resolution crystal structure of a ternary complex of PolC with DNA and incoming deoxynucleoside triphosphate (dNTP). However, kinetic information needed to understand the enzymatic mechanism of C-family polymerases is limited. For this study we have performed a detailed steady-state and pre-steady-state kinetic characterization of correct dNTP incorporation by PolC from the Gram-positive pathogen *Staphylococcus aureus*, using a construct lacking both the non-conserved N-terminal domain and the 3′–5′ exonuclease domain (*Sau*-PolC-ΔNΔExo). We find that *Sau*-PolC-ΔNΔExo has a very fast catalytic rate (k_pol_ 330 s^−1^) but also dissociates from DNA rapidly (k_off_ ∼150 s^−1^), which explains the low processivity of PolC in the absence of sliding clamp processivity factor. Although *Sau*-PolC-ΔNΔExo follows the overall enzymatic pathway defined for other polymerases, some significant differences exist. The most striking feature is that the nucleotidyl transfer reaction for *Sau*-PolC-ΔNΔExo is reversible and is in equilibrium with dNTP binding. Simulation of the reaction pathway suggests that rate of pyrophosphate release, or a conformational change required for pyrophosphate release, is much slower than rate of bond formation. The significance of these findings is discussed in the context of previous data showing that binding of the β-clamp processivity factor stimulates the intrinsic nucleotide incorporation rate of the C-family polymerases, in addition to increasing processivity.

## Introduction

DNA replication is the complex process of genome duplication involving several different proteins that form the “replisome”. A key enzyme of the replisome is the DNA polymerase, a nucleotidyl transferase that catalyzes the addition of a deoxynucleoside triphosphate (dNTP) to the nascent DNA chain. All organisms have several types of DNA polymerases of which the ones responsible for duplicating most of the genome are known as replicative polymerases. These are characterized by being highly efficient enzymes that can select the next correct nucleotide with extraordinarily high accuracy in a template-dependent manner.

The replicative polymerases of all bacteria are grouped by sequence similarity into the C-family of DNA polymerases [1], [2], but subdivide into two branches. The *polC* gene encodes the replicative polymerase of Gram-positive bacteria with low G+C content, while the *dnaE* gene encodes for the same in Gram-negative bacteria and in Gram-positive bacteria with high G+C content [3]. The replisomes of both Gram-positive and Gram-negative bacteria have been reconstituted and studied *in vitro* providing a wealth of knowledge about how replication occurs inside the bacterial cell [4]–[15].

**Figure 1:**
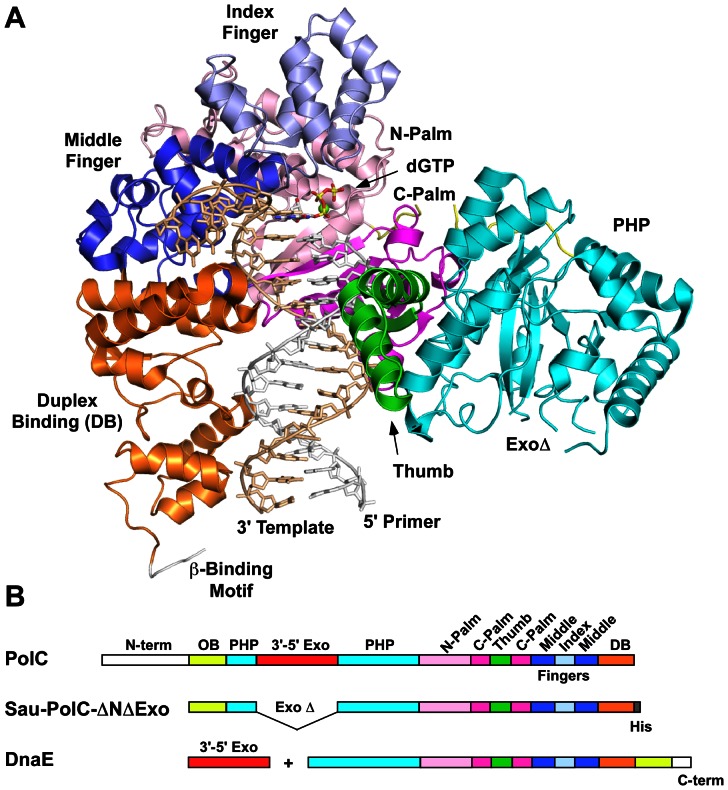
Overview of PolC structure and domain organization. A. Structure of *Gka-*PolC-ΔNΔExo shown (PDB ID: 3F2B). Domains are color coded as in Figure 1B. OB domain is not shown for simplicity. The linker connecting the N-palm and PHP domains is shown (yellow). B. Sequence alignment showing domain organization of C-family polymerases.

Recently, several crystal structures of C-family polymerases have been reported, including DnaE from *Escherichia coli* and *Thermus aquaticus* and PolC from *Geobacillus kaustophilus* [16]–[19]. Other than non-conserved N- and C-terminal extensions, the individual domains of PolC and DnaE are structurally conserved [17], as expected from sequence conservation, but differ somewhat in their linear organization in the protein (Figure 1). The OB domain of PolC is located just after the non-conserved N-terminal domain, while the OB domain of DnaE is located just before the non-conserved C-terminal domain. Additionally, PolC contains an intrinsic 3′ to 5′ exonuclease proofreading domain that is absent in DnaE. In Gram-negative bacteria, this function is performed by the epsilon (ε) subunit [20] of the replisome, which is homologous to the PolC exonuclease domain. A remarkable finding from the crystallographic studies is that the bacterial replicative polymerases are not related to the replicative polymerases from either eukaryotes or the archaea.

Despite the fact that *in vitro* studies of the bacterial replisome have been key to delineating the main features of DNA replication in all forms of life [20], comparatively little is known about the kinetic mechanism of polymerization by C-family polymerases. This contrasts with the extensive kinetic information available for other polymerase families, including the B-family enzymes that are the replicative polymerases in eukaryotes and most archaea. Thus, the foundation for detailed structure-function studies of C-family polymerases has not yet been laid.

**Figure 2:**
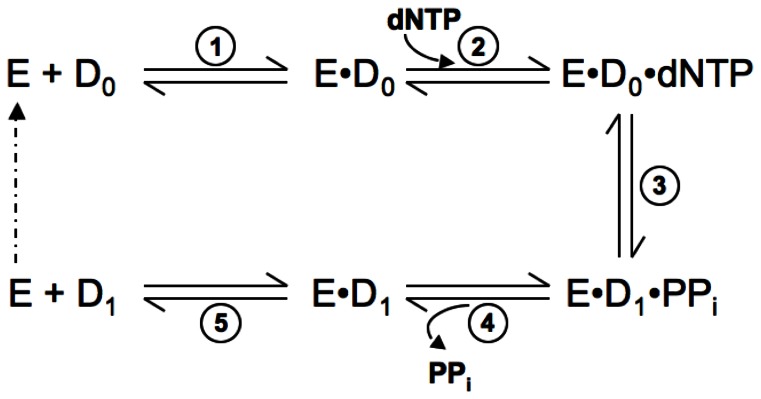
Minimal single-nucleotide incorporation reaction pathway for DNA polymerases. Abbreviations used were: E, DNA polymerase; D_0_, unextended DNA; D_1_, DNA extended by one base-pair; PPi, inorganic pyrophosphate. Dashed arrow indicates the polymerase entering another round of catalysis.

For all polymerases studied to date, the same overall enzymatic pathway (Figure 2) has been established for correct nucleotide incorporation [21]–[25]. The minimal pathway involves substrates binding to the polymerase in an ordered manner, with DNA binding first (step 1), followed by binding of the incoming dNTP (step 2). This is succeeded by the chemical step of bond formation (step 3). Typically, this step is preceded by a slower step along the pathway, which has been interpreted as a conformational change of the polymerase [21], [23]. Earlier structural studies suggested that this slow step might correspond to the large-scale domain movement associated with nucleotide binding [26], [27], but more recent studies have shown that motion to be too fast to be rate limiting [28], [29]. Although the conformational change accompanying nucleotide binding is faster than chemistry, it still controls specificity of nucleotide addition [30], [31]. Currently, the slower conformational change is thought to be a later, smaller-scale movement, but the precise nature of this non-covalent change is not known and may vary among different polymerases. Bond formation is followed by the release of the pyrophosphate (PPi) (step 4) generated during phosphoryl transfer. This step is generally presumed to be rapid [21] and is likely to be accompanied by the reverse of the conformational change that is induced by dNTP binding. Finally, release of the product DNA occurs (step 5), allowing the polymerase to perform subsequent rounds of catalysis. During processive synthesis, DNA would translocate along the polymerase rather than dissociate.

In this study we have performed both steady-state and pre-steady-state kinetic characterization of correct dNTP incorporation by an N-terminal and exonuclease domain deficient mutant of *Staphylococcus aureus* PolC (*Sau*-PolC-ΔNΔExo). This construct has the identical domain organization as the *G. kaustophilus* PolC used in the crystallographic study (*Gka*-PolC-ΔNΔExo) [17]. Furthermore, this construct has all the domains that are conserved between the PolC and DnaE polymerases (Figure 1). Hence, kinetic data obtained using this construct can be utilized directly for making structure-function correlations among the C-family polymerases and establish a foundation for future mechanistic studies of this polymerase family.

## Materials and Methods

### Materials

5′-6FAM labeled primer and unlabeled template oligonucleotides were purchased from Integrated DNA Technologies, Inc. Unlabeled ultrapure grade dTTP was purchased from GE Healthcare Biosciences. All the graphs and nonlinear regressions were done using GraphPad Prism, version 6.0a (GraphPad Software Inc.). Simulation of the reaction mechanism of *Sau*-PolC-ΔNΔExo was performed using KinTek Explorer, version 3.0 (KinTek Corp.) [32], [33].

### Duplex DNA Formation

The primer and template DNA oligonucleotides were incubated together in annealing buffer (10 mM Tris-HCl (pH 8), 1 mM EDTA and 100 mM NaCl) and heated to a temperature of 95°C followed by gradual cooling to room temperature.

### Expression and Purification of Sau-PolC-ΔNΔExo

*S. aureus* PolC lacking the N-terminal domain (amino acids 1–207) and the exonuclease domain (amino acids 415–609) and containing a C-terminal hexahistidine tag (*Sau*-PolC-ΔNΔExo) was expressed from a pET32A vector and was a generous gift from Thale Jarvis (Crestone Inc.). The plasmid was transformed into Rosetta(DE3)pLysS *E. coli* cells. Cells were grown to an OD_600_ of ∼0.65 and then induced with 0.5 mM IPTG for ∼16 hrs at 17°C. All subsequent steps were carried out at 4°C. Cell pellets were resuspended in IMAC buffer (50 mM Tris-HCl (pH 7.5), 800 mM NaCl, 10 mM imidazole and 10% glycerol). In order to prevent proteolytic degradation of *Sau*-PolC-ΔNΔExo, EDTA-free protease inhibitor tablet (Roche) was added to IMAC buffer at a concentration of 1 tablet/10 g of cells. Cells were lysed by sonication and the clarified cell lysate was passed through Ni-NTA columns (3×5 ml). In order to reduce the NaCl concentration to 100 mM for later steps, the columns were washed with 10 column volumes of low salt IMAC buffer (50 mM Tris-HCl (pH 7.5), 100 mM NaCl, 10 mM imidazole and 10% glycerol). The protein was eluted using a linear gradient of imidazole from 10 to 400 mM in low salt IMAC buffer. During elution, two proteins with molecular weights of ∼75 kD eluted before *Sau*-PolC-ΔNΔExo. These are likely to be partial proteolytic products of *Sau*-PolC-ΔNΔExo and care was taken to remove these contaminants during elution from the Ni-NTA columns. Intact *Sau*-PolC-ΔNΔExo obtained from Ni-NTA chromatography was loaded onto Q-sepharose columns (HiTrap Q XL, 2×5 ml) pre-equilibrated in Buffer A (50 mM Tris-HCl (pH 7.5), 100 mM NaCl, 5 mM EDTA, 10% glycerol and 1 mM DTT). The protein was eluted from the Q column using a linear gradient of NaCl from 100 mM to 1 M in Buffer A. Eluent of the Q column was diluted ∼7 fold in Buffer A, to a NaCl concentration of ∼100 mM, and was subjected to heparin column chromatography (HiTrap Heparin HP, 2×5 ml). Buffer A was used to pre-equilibrate the heparin columns and protein was eluted using a linear gradient of NaCl ranging from 100 mM to 1 M in Buffer A. As a final step of purification, *Sau*-PolC-ΔNΔExo eluted from the heparin column was subjected to size exclusion chromatography using a Superdex 200 column (HiLoad 16/60 Superdex 200 pg) pre-equilibrated in storage buffer (50 mM Tris-HCl (pH 7.5), 250 mM NaCl, 5 mM EDTA, 10% glycerol and 1 mM DTT). The purified protein obtained from the size exclusion column was concentrated to ∼150–200 µM, calculated from the OD_280_ of the protein sample using a theoretical extinction coefficient of 87,100 M^−1^cm^−1^, and was stored at −80°C.

### Assays for Optimization of Enzymatic Activity of Sau-PolC-ΔNΔExo

Primer extension assays were performed in order to determine the optimum buffer conditions for the enzymatic activity of *Sau*-PolC-ΔNΔExo. 400 nM p/t DNA was incubated with 1 nM *Sau*-PolC-ΔNΔExo in PolC reaction buffer (25 mM MES-Tris (pH 8), 25 mM NaCl, 8 mM MgCl_2_, 2 mM DTT and 5% glycerol). All the components of the PolC reaction buffer were kept fixed except the component whose effect was being tested. Unless mentioned otherwise, all the assays were carried out at room temperature (25°C). Assays were initiated by addition of 1 mM (final concentration) dTTP to the reaction mix. After 2 minutes, an equal volume of 250 mM EDTA was added to quench the assays. The extended and unextended primers were separated on a 17% acrylamide/7 M urea denaturing 1xTBE gel. The gel was imaged using a Typhoon 9400 scanner (GE Healthcare) and bands were quantitated using ImageQuant software (GE Healthcare). Percentage of primer extension was determined by measuring the relative intensity of the band corresponding to the extended primer with respect to the total labeled DNA (i.e. both extended and unextended primer strands). All reactions were performed in triplicate.

### Steady-state Assays

Primer extension assays done for determining the steady-state parameters were performed using a KinTek RQF-3 rapid quench instrument (KinTek Corp.). Reactions were initiated by mixing a pre-equilibrated solution of 5 µM p/t DNA and 50 nM total *Sau*-PolC-ΔNΔExo in PolC reaction buffer (this corresponded to an active enzyme concentration of 33 nM, as described in the “Active site titration” section of the Results) to an equal volume of various concentrations of dTTP (18.76 to 600 µM) in the same buffer. Hence, the final p/t DNA and active *Sau*-PolC-ΔNΔExo concentrations in the reactions were 2.5 µM and 16.5 nM respectively and the final concentration range of dTTP was 9.38 to 300 µM. The assays were quenched at various time intervals by addition of 250 mM EDTA. The time intervals were adjusted such that primer extension was between 5–15%. Separation and quantitation of the extended primers was performed as described above. The concentration of primers extended for different concentrations of dTTP were plotted as a function of time and the data were fit to the steady-state rate equation:
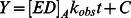
(1)where Y is the concentration of primer extended, [ED]_A_ is the concentration of active *Sau*-PolC-ΔNΔExo ⋅ p/t DNA binary complex that gets converted to product, k_obs_ is the observed rate of primer extension, t is time interval after which the reaction was quenched, and C is a constant. The observed rates were plotted as a function of dTTP concentration ([dTTP]) and the data were fit to the Michaelis-Menten equation:
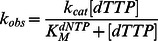
(2)where k_cat_ is the maximum steady-state rate and KM is the Michaelis constant for dNTP.

### Measurement of DNA Dissociation Rate from Binary Complex

Kintek RQF3 rapid quench device was used to perform this experiment. 300 nM *Sau*-PolC-ΔNΔExo (this corresponded to an active enzyme concentration of 200 nM, as described in the “Active site titration” section of the Results) was preincubated with 160 nM p/t DNA in PolC reaction buffer in a 16 µl reaction volume. This was mixed with an equal volume of 96 µM unlabelled p/t DNA in the same buffer and incubated for various time intervals (0.005–0.05 s). Finally, ∼80 µl of 200 µM dTTP was added for primer extension (∼140 µM final concentration). At this stage the reaction was allowed to proceed for 0.028 s and quenched by collection of the sample in a microfuge tube containing 100 µl of 250 mM EDTA. Concentration of the extended primer was plotted as a function of time. Data were fit to the following exponential equation (Equation 3) and the rate of decrease of product formation was interpreted as the rate of dissociation of *Sau*-PolC-ΔNΔExo from the preformed *Sau*-PolC-ΔNΔExo ⋅ p/t DNA binary complex
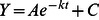
(3)where, Y is the concentration of the product formed, A is the amplitude, k is the rate of product formation, t is the first incubation time (ranging from 0.005 to 0.05 s) and C is a constant. Reactions were performed in triplicate.

### Active Site Titration of Sau-PolC-ΔNΔExo

300 nM *Sau*-PolC-ΔNΔExo was pre-equilibrated with various concentrations of p/t DNA (20 to 1800 nM) in PolC reaction buffer. The reactions were initiated by rapid mixing of this solution with an equal volume of PolC reaction buffer containing 2 mM dTTP in a KinTek RQF-3 rapid quench instrument. Final concentrations were 10–900 nM p/t DNA, 150 nM polymerase and 1 mM dTTP. The reactions were terminated at different time intervals by addition of 250 mM EDTA. The time-courses of primer extension for different p/t DNA concentrations were fit to the full burst equation:
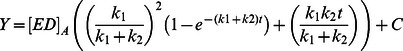
(4)where Y is the concentration of the extended primer, [ED]_A_ is the concentration of the preformed active enzyme ⋅ DNA binary complex that gets converted to product before turnover, k_1_ is the rate of the fast phase, k_2_ is the rate of the slow phase, t is the time interval after which the reaction was quenched and C is a constant. [ED]_A_ for different DNA concentrations thus obtained were plotted out as a function of p/t DNA concentration and fit to the following quadratic equation:
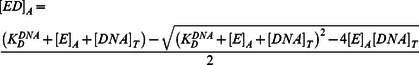
(5)where KDDNA is the dissociation constant for binding of Sau-PolC-ΔNΔExo to p/t DNA, [E]A is the concentration of active Sau-PolC-ΔNΔExo and [DNA]T is the concentration of total p/t DNA at the beginning of the assay.

### K_D_^dNTP^ Determination

Primer extension assays were performed with a RQF-3 rapid quench instrument using final concentrations of 804 nM active *Sau*-PolC-ΔNΔExo, 50 nM p/t DNA and various [dTTP] (1.17 to 100 µM). Reactions were quenched by addition of 250 mM EDTA. Time courses of primer extension reactions were plotted as a function of [dTTP] and the data were fit to the full burst equation (Equation 4). The rate, k_1_, and [ED]_A_ thus obtained were further plotted against [dTTP] and then fit to the appropriate hyperbolic equation:
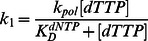
(6)

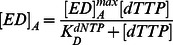
(7)where k_pol_ is the maximum rate of the burst of product formation, K_D_^dNTP^ is the dissociation constant for dNTP binding to the *Sau*-PolC-ΔNΔExo ⋅ p/t DNA binary complex and [ED]_A_^max^ is the maximum concentration of the enzyme ⋅ DNA binary complex that gets converted to product before turnover.

Unless mentioned otherwise, all reactions were done in at least three independent experiments, using two different preparations of *Sau*-PolC-ΔNΔExo. All data were combined and analyzed together.

### Simulation

The reaction mechanism of *Sau*-PolC-ΔNΔExo was simulated using KinTek Explorer software. Details about the mechanism used for the simulation is discussed under “Results” section. The software was used to fit data to the simulation using an iterative procedure until a “best fit” was achieved. The simulated curves and the raw data were exported from the software and final plots overlaying the raw data with the simulated curves were prepared using GraphPad Prism. To determine the range within which each of the rate constants was constrained by the model, and to investigate the relationships between different rate contants, we computed 3-D confidence contour plots for all possible pairs of rate constants. See reference [32] for a detailed description of how to interpret these plots.

## Results

### PolC Purification

**Figure 3:**
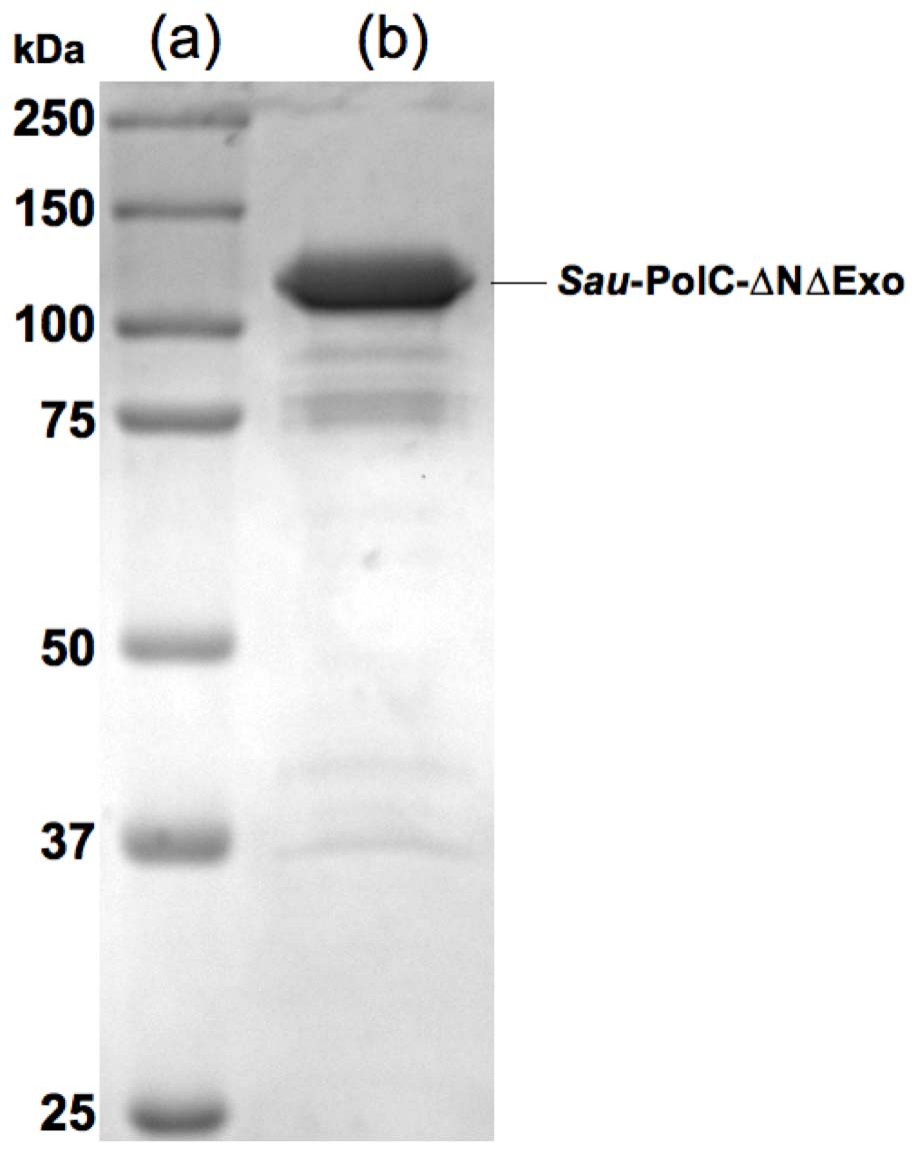
SDS-PAGE of Sau-PolC-ΔNΔExo. A 10% SDS-polyacrylamide gel stained with Coomassie R-250 showing purified *Sau*-PolC-ΔNΔExo obtained after size exclusion chromatography. (a) Kaleidoscope pre-stained marker. (b) 2.5 µM purified *Sau*-PolC-ΔNΔExo.

Recombinant *Sau*-PolC-ΔNΔExo was purified using Ni-NTA, anion exchange, heparin and size-exclusion chromatography. Figure 3 shows SDS-PAGE analysis of the final purified protein. *Sau*-PolC-ΔNΔExo migrates as expected for a protein with a theoretical molecular weight of 120 kD. As has been noted previously for full-length PolC [12], inducing protein expression at temperatures below 20°C was critical for obtaining purified protein, estimated to be ∼95% homogeneous, that did not have significant levels of proteolytic products.

### Optimal Reaction Conditions

**Figure 4:**
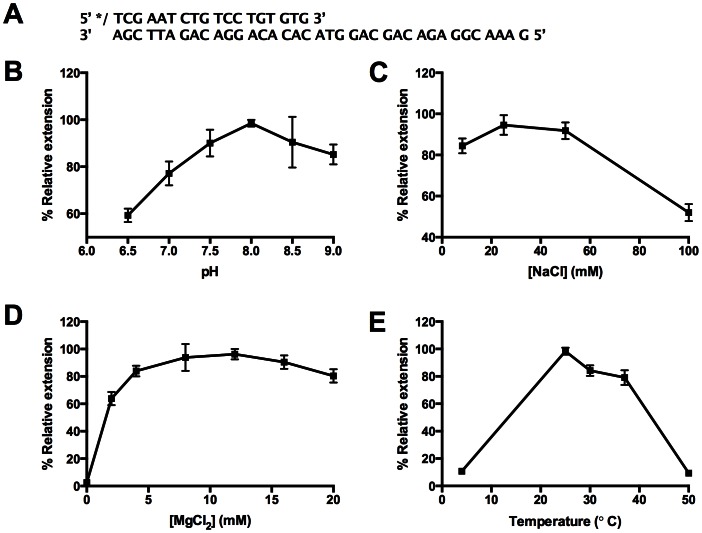
Primer extension assays for optimizing enzymatic activity of Sau-PolC-ΔNΔExo. (**A**) Duplex DNA sequence used for all primer extension assays performed in this study. “*/” at 5′ end of primer indicates 6-FAM label. (**B**) Effect of pH (**C**) Effect of NaCl concentration (**D**) Effect of Mg^2+^ concentration and (**E**) Effect of temperature on *Sau*-PolC-ΔNΔExo activity. Primer extension assays were carried out under steady-state conditions by adding 1 mM dTTP (the correct incoming dNTP) to a pre-incubated solution of 400 nM p/t DNA and 1 nM *Sau*-PolC-ΔNΔExo. Reactions were quenched after 2 minutes by addition of an equal volume of 250 mM EDTA. Unextended and extended primers were separated by gel electrophoresis on a 17% denaturing TBE-acrylamide gel. Fraction of primer DNA extended was determined by measuring the relative intensity of the extended primer band with respect to the total labeled DNA (extended and unextended primer).

Reaction conditions for *Sau*-PolC-ΔNΔExo were optimized by quantitating incorporation of the next correct dNTP on a p/t DNA with an 18-bp duplex region and a 19-nt single stranded template region (Figure 4A). All reaction conditions were kept constant, except for the one whose effect was being tested. The reaction conditions varied were: pH of the buffer, concentration of NaCl, concentration of Mg^2+^ and reaction temperature (Figure 4B–E). Dependence of primer extension on the buffer pH followed a bell shaped curve typical of an acid-base reaction, with an optimum pH of 8 (Figure 4B). The rate of primer extension was found to decrease with an increase in the concentration of NaCl, with the maximum activity occurring at 25 mM NaCl (Figure 4C). A concentration of 8 to 12 mM Mg^2+^ was found to be optimal for enzymatic activity of *Sau*-PolC-ΔNΔExo (Figure 4D). No primer extension was observed in the absence of Mg^2+^, as expected for a polymerase using a two-metal-ion mechanism. Very little primer extension occurred at 4°C and 50°C, but, for all other temperatures tested (25°C, 30°C and 37°C), the enzyme performed well (Figure 4E). Based on these results, all subsequent reactions were performed at 25°C at pH 8 with 25 mM NaCl and 8 mM Mg^2+^.

### Michaelis-Menten Kinetics

**Figure 5:**
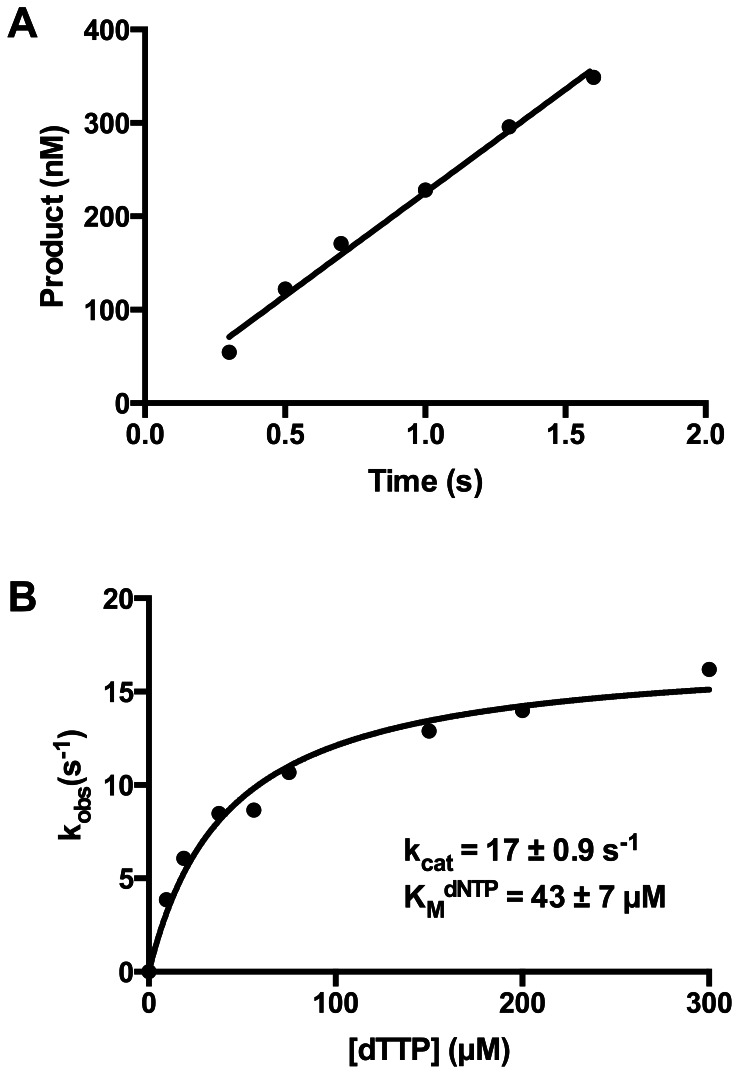
Steady-state kinetic analysis of Sau-PolC-ΔNΔExo. Primer extension assays were performed by adding dTTP (final concentration range 9.38 to 300 µM) to a final concentrations of 2.5 µM p/t DNA and 16.5 nM active *Sau*-PolC-ΔNΔExo. The reactions were quenched at different time intervals with 250 mM EDTA. (**A**) A typical time course of primer extension followed during the steady-state kinetic assays (final [dTTP] was 300 µM). The concentration of primer extended was plotted against time and fit to the steady-state equation (Equation 1). (**B**) Michaelis-Menten plot for *Sau*-PolC-ΔNΔExo. The observed rates of primer extension were plotted as a function of the dTTP concentration. The resulting plot was fit to the Michaelis-Menten equation (Equation 2). From the fit, steady-state rate constant (k_cat_) was calculated to be 17±1 s^−1^ and Michaelis constant for dNTP (K_M_^dNTP^) was determined to be 43±7 µM.

Primer extension assays were performed under steady-state conditions, with substrates present in excess of enzyme, using a final concentration of 2.5 µM p/t DNA, 16.5 nM active *Sau*-PolC-ΔNΔExo (see result of “Active site titration” for calculation of active enzyme concentration) and various concentrations of dTTP ranging from 9.38 to 300 µM. Observed rates of nucleotide incorporation were calculated from the concentration of product formed vs. time, using the linear portion of progress curves (Figure 5A), and were plotted as a function of [dTTP] (Figure 5B). The data were fit to the Michaelis-Menten equation (Equation 2) and gave a k_cat_ of 17±1 s^−1^ and K_M_^dNTP^ of 43±7 µM.

### Rate of Dissociation of p/t DNA from the Binary Complex

**Figure 6:**
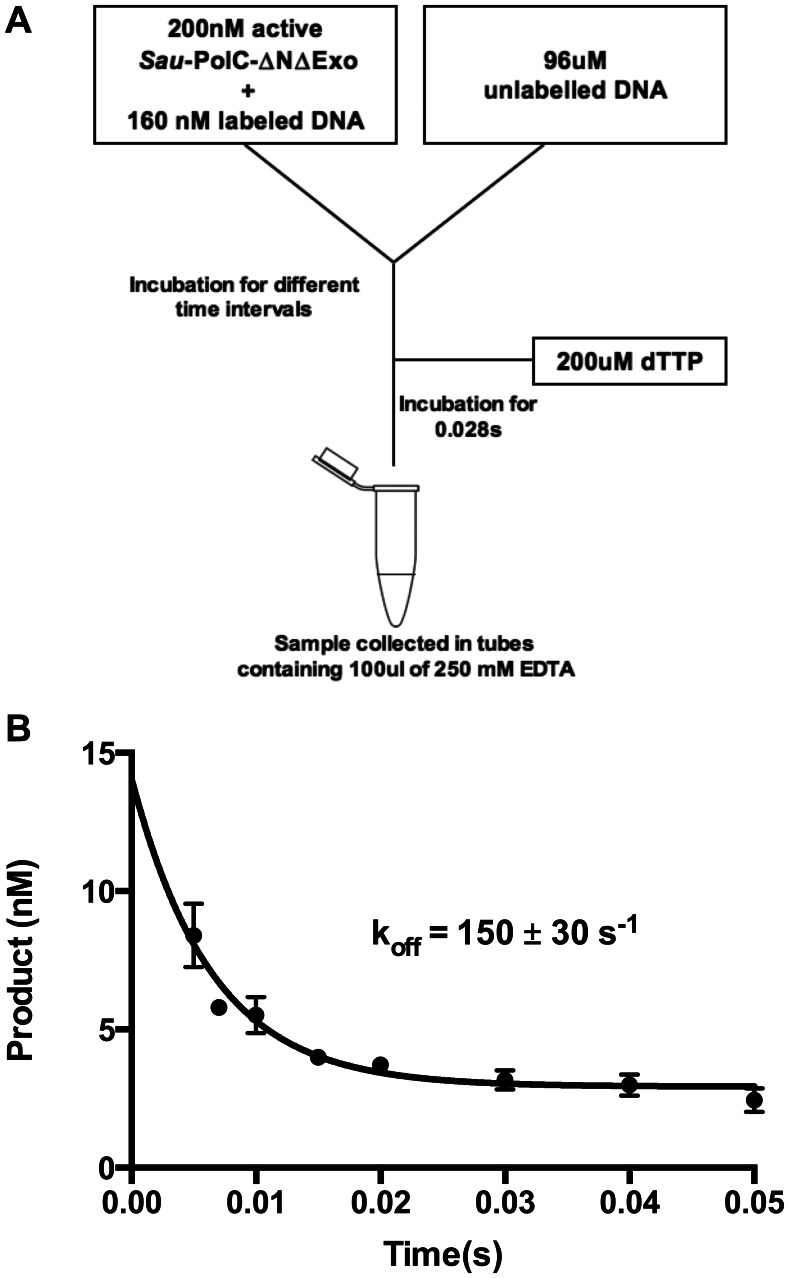
Determination of the DNA dissociation rate from polymerase ⋅ DNA binary complex (k_off_). (**A**) Schematic representation of the experimental procedure. (**B**) Plot of product formed vs time. The data were fit to a single exponential equation (Equation 3). The rate of decrease in product formation (which is equivalent to the rate of dissociation of p/t DNA from *Sau*-PolC-ΔNΔExo ⋅ p/t DNA binary complex (k_off_)) was 150±30 s^−1^.

Typically, in single-nucleotide incorporation assays such as the one used here, dissociation of the DNA from the polymerase ⋅ DNA binary complex is the rate limiting step of the catalytic cycle, and the rate of this step (k_off_) governs the steady-state rate (k_cat_). To determine if this was the case for *Sau*-PolC-ΔNΔExo, we directly measured k_off_ using the experimental design shown in Figure 6A. For this experiment *Sau*-PolC-ΔNΔExo was preincubated with p/t DNA and the preformed *Sau*-PolC-ΔNΔExo ⋅ p/t DNA binary complex was rapidly mixed with an equal volume of unlabelled p/t DNA. The resulting reaction (containing final concentrations of 150 nM *Sau*-PolC-ΔNΔExo, 80 nM p/t DNA, and 48 µM unlabelled p/t DNA) was incubated for various time intervals ranging from 0.005 to 0.05 s. The unlabelled p/t DNA trapped any *Sau*-PolC-ΔNΔExo that dissociated from the preformed binary complex during this time. Next, dTTP was added to initiate the reaction and a further incubation of 0.028 s was performed, to allow extension of p/t DNA bound to PolC. Finally, the reaction was quenched with EDTA. A plot of labeled product formed versus the variable incubation time (0.005 to 0.05 s) showed a clear reduction in product concentration with increasing time (Figure 6B). This was attributed to the decrease in the concentration of the preformed *Sau*-PolC-ΔNΔExo ⋅ p/t DNA due to the dissociation of the labeled p/t DNA from the complex and rebinding of the enzyme to the excess unlabeled p/t DNA. The data fitted well to an exponential equation (Equation 3) and the rate of decrease in product formation (k_off_) was found to be 150±30 s^−1^. This indicated that for *Sau*-PolC-ΔNΔExo, DNA dissociation is approximately 9-fold faster than k_cat_ and, surprisingly, is not the rate-limiting step of the of the single-nucleotide incorporation cycle.

### Pre-steady-state Burst Kinetics

**Figure 7:**
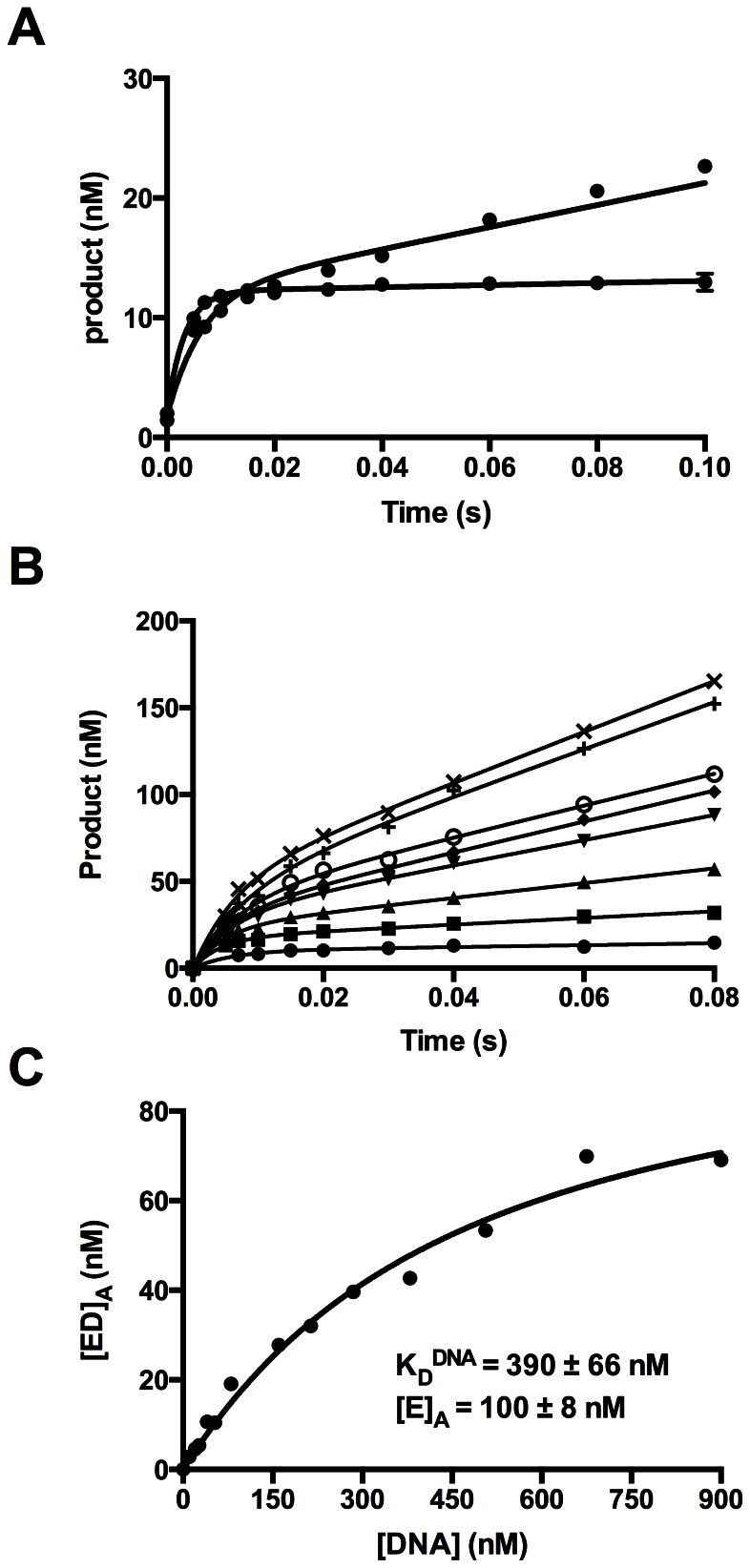
Pre-steady-state kinetics and active site titration of Sau-PolC- ΔNΔExo. (**A**) A time course of primer extension under pre-steady-state condition in the presence (⋅) and absence (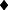) of unlabelled p/t DNA acting as an enzyme trap. 35 µM dTTP (with or without 48 µM of unlabelled p/t DNA) was added to 150 nM *Sau*-PolC-ΔNΔExo (corresponding to an active *Sau*-PolC-ΔNΔExo concentration of 100 nM) and 80 nM p/t DNA (all concentrations are final). In the absence of the trap, the time course was biphasic in nature and the data were fit to the full burst equation (Equation 4). The rate of the fast phase was 150**±**30 s^−1^ and that of the slower phase was 8.5**±**1 s^−1^, [ED]_A_ was found to be 12**±**1 nM. In the presence of the trap, the time course was monophasic and the data were fit to a single exponential equation, with a rate of 300**±**14 s^−1^ and an amplitude of 11.5**±**0.5 nM. The data can also be fit equally well to the full burst equation, but the data were not sufficient to justify using the more complex model. (**B**) A representative set of primer extension assays performed during active site titration. Time resolved primer extension assays were performed using 150 nM *Sau*-PolC-ΔNΔExo, 1 mM dTTP and varying concentrations of p/t DNA (⋅ 40 nM, ▪ 80 nM, 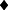 160.1 nM,♦ 284.76 nM,<$>\raster="rg1"<$> 379.69 nM, ○ 506.25 nM, **+**675 nM and ×900 nM). The concentration of extended primer was plotted versus time and data were fit to the full burst equation (Equation 4). For ease of understanding, the background primer extension has been deducted from each time course. (**C**) A plot of the concentrations of pre-formed active enzyme-DNA complex getting converted to product before turnover ([ED]_A_) versus DNA concentration was fit to a quadratic equation (Equation 5). K_D_^DNA^ was determined to be 390±70 nM and the concentration of active *Sau*-PolC-ΔNΔExo was found to be 100±8 nM.

To determine if *Sau*-PolC-ΔNΔExo displayed a rate-limiting step after chemistry, primer extension assays were performed under pre-steady-state conditions with a total enzyme concentration of 150 nM and 80 nM p/t DNA (final concentrations). After pre-incubation to form the binary complex, reactions were started by the addition of dTTP to a final concentration of 35 µM and product formation was followed up to 0.2 s. Plot of the concentration of product formed with respect to time was biphasic in nature (Figure 7A). The fast phase represents the initial burst of dTTP incorporation by the pre-formed *Sau*-PolC-ΔNΔExo ⋅ p/t DNA binary complex, while the slow phase represents dTTP incorporation in subsequent rounds of primer extension, after the enzyme dissociates from the first p/t DNA substrate and rebinds another. The data were fit using the full burst equation (Equation 4) [34]. The rates of the fast and slow phases obtained were 150±30 s^−1^ and 8.5±1 s^−1^, respectively. Product formed during the fast burst phase was 12±1 nM, indicating that out of 150 nM of *Sau*-PolC-ΔNΔExo, only 12 nM formed active enzyme ⋅ DNA binary complex that got converted to product.

Since the rate of dissociation of the DNA substrate from the binary complex (Figure 2, step 5) was very fast, it was possible that the DNA did not form a stable ternary complex even in the presence of the correct incoming dNTP (Figure 2, step 3). In order to test whether such was the case, we repeated the above burst experiment in the presence of 48 µM of unlabelled p/t DNA that was added at the same time as the dTTP. Any *Sau*-PolC-ΔNΔExo that dissociated from the labeled p/t DNA would be trapped by the excess unlabelled DNA, which would eliminate the slower phase. Additionally, any unstable ternary complex having a dissociation rate comparable to the rate of chemistry or faster would result in lower amplitude of product formation in the presence of the trap. Our result shows that, in the presence of the DNA trap, the slow phase was eliminated, as expected, and the amplitude of product formation was 11.5±0.5 nM (Figure 7A), identical to the amplitude in the absence of the trap.

These results indicate that although DNA dissociation from the binary complex is rapid, disassembly of the ternary complex is not rapid and, during a single nucleotide-incorporation cycle, DNA does not dissociate from the enzyme after nucleotide binds. The difference in the rates of product formation for the first and subsequent rounds of enzyme turnover, as observed in the burst experiment, indicates the presence of a slow and at least partially rate-limiting step after dNTP incorporation. The low burst amplitude suggested that binding of *Sau*-PolC-ΔNΔExo to p/t DNA was weak and/or only a fraction of the enzyme was active. A third possibility is the presence of an internal equilibrium in the pathway leading to a reduction in product formation. The following experiments indicate that all three of these possibilities contribute to the low burst amplitude.

### Active Site Titration

The formation of a stable ternary complex and the presence of a slow, rate-limiting step after chemistry allowed us to perform burst kinetic assays to determine the apparent K_D_^DNA^ and the concentration of active *Sau*-PolC-ΔNΔExo. For these assays, the final concentration of total *Sau*-PolC-ΔNΔExo was 150 nM and the final DNA concentration was varied between 10 and 900 nM. Product formation for a representative set of DNA concentrations is shown in Figure 7B. The time courses were fit to the full burst equation, and the concentrations of the initial active enzyme ⋅ DNA complex that was converted into product during the first round of catalysis ([ED]_A_), obtained from the amplitudes of the fast phase, were plotted as a function of DNA concentration (Figure 7C). The data were fit to a quadratic equation (Equation 5). From the fit, the apparent K_D_^DNA^ was determined to be 390±70 nM, indicating a relatively weak binding to DNA (Table 1), and the concentration of active *Sau*-PolC-ΔNΔExo was 100±8 nM, implying that ∼70% of the *Sau*-PolC-ΔNΔExo was active. The active enzyme concentration was lower than expected given the purity of the preparation, but the result was consistent for different preparations. From the apparent DNA binding affinity and the rate of DNA dissociation, we estimate that *Sau*-PolC-ΔNΔExo associates with DNA with a rate constant (k_on_) of ∼4×10^8^ M^−1^s^−1^, which suggests that the rate of DNA binding is limited by diffusion.

**Table 1:** Comparison of kinetic parameters of different polymerases.

Polymerase	Family	K_D_^DNA^ (nM)	K_D_^dNTP^ (µM)	k_pol_ (s^−1^)	k_off_ † (s^−1^)	References
***Sau-PolC-***Δ***N***Δ***Exo (observed)***	**C**	**390**	**4**	**180**	**150**	**–**
***Sau-PolC-***Δ***N***Δ***Exo (simulated)***	**C**	**–**	**7.5**	**330**	**–**	**–**
Pol I (Klenow)	A	5	5.5	50	0.06	[24]
*T7DNA Polymerase*	A	(18)	(18)	(287)	(0.2)	[25]
E. coli Pol II	B	21	4.4	13	0.05	[48]
*Mammalian Polδ*	B	300	**–**	7	0.005	[49]
		(64)	(0.93)	(21)	(0.006)	
*Yeast Polδ*	B	30	24	1	0.045	[50]
*Human mitochondrial Polγ*	B	39	14	3.5	0.03	[51], [52]
		(9.9)	(0.78)	(45)	(0.02)	
Polβ	X	49	110	10	0.3	[53]
E. coli Pol IV	Y	50	441	12	0.18	[54]
		**–**	(51)	(22)	(0.03)	

Data obtained from this study are highlighted in bold. Replicative polymerases are shown in italics. Values shown in parentheses were measured in the presence of the corresponding processivity factor.

† For all DNA polymerases shown here except for *Sau-PolC-*Δ*N*Δ*Exo* k_off_ is equivalent to k_cat._

### Nucleotide Binding Affinity

**Figure 8:**
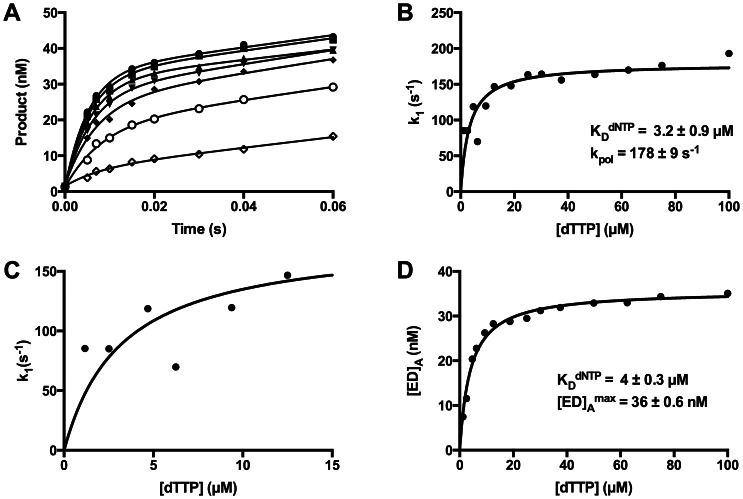
Determination of K_D_^dNTP^ of Sau-PolC- ΔNΔExo. (**A**) A representative set of primer extension assays performed during K_D_^dNTP^ determination for *Sau*-PolC-ΔNΔExo. The reactions were performed with 804 nM active *Sau*-PolC-ΔNΔExo, 50 nM p/t DNA and various concentrations of dTTP (◊ 1.17 µM, ○ 4.69 µM, ♦ 9.4 µM, ▾ 18.75 µM, ▴ 30.14 µM, ▪ 50 µM and • 75 µM). The concentrations of extended primers were plotted against time and the plots were fit to the full burst equation (Equation 4). (**B**) A plot of the observed rates of the fast phase (k_1_) versus [dTTP]. The data were fit to a hyperbolic equation (Equation 6). From the fit, K_D_^dNTP^ was determined to be 3.2±0.9 µM and maximum rate of the burst (k_pol_) was found to be 178±9 s^−1^. R^2^ value for this fit was 0.75. (C) An enlarged view of panel (B) of up to 15 µM of [dTTP]. (D) A plot of [ED]_A_ versus [dTTP] was fit to a hyperbolic equation (Equation 7). From the fit, K_D_^dNTP^ was found to be 4.0±0.3 µM and the maximum [ED]_A_ was 36±0.6 nM. R^2^ value for this fit was 0.98.

Primer extension assays were again performed under burst conditions to determine the apparent K_D_^dNTP^ and k_pol_. For these assays the final concentrations of active *Sau*-PolC-ΔNΔExo and p/t DNA were 804 nM and 50 nM respectively. The concentration of dTTP was varied from 1.17 to 100 µM and a representative range of data is shown in Figure 8A. Rates of the fast phase (k_1_), obtained by fitting the time course to the full burst equation, were plotted against [dTTP] and the data were fit to a hyperbolic equation (Equation 6). From the fit, the apparent K_D_^dNTP^ was determined to be 3.2±0.9 µM and k_pol_ was 180±9 s^−1^ (Figure 8B). Although these parameters were reasonable compared to other replicative polymerases, the overall fit to the data was not good (R^2^ of 0.75), primarily because the observed rates for lower nucleotide concentrations did not match well with the rates predicted by the hyperbolic equation (Figure 8C). The deviation of the observed nucleotide incorporation rates appeared to be due to the lower amplitudes of the fast phase at low nucleotide concentrations.

### Dependence of Active Enzyme⋅DNA Binary Complex on [dNTP]

Typically, bond formation (Figure 2, step 3) is irreversible, because pyrophosphate release (Figure 2, step 4) is fast, and the binding of the incoming dNTP is a rapid equilibrium process [21]. Hence there is no equilibrium between dNTP binding (Figure 2, step 2) and bond formation. Therefore, an increase in the concentration of the incoming dNTP does not influence the concentration of the preformed active enzyme ⋅ DNA binary complex that gets converted to product before turnover ([ED]_A_). This is observed as the lack of correlation in a plot of [ED]_A_ versus [dNTP]. Upon closer inspection of our data, however, we observed that [ED]_A_ obtained from the burst amplitude of the fast phase was dependent on the dTTP concentration, saturating at higher concentrations (Figure 8D) and fit well (R^2^ of 0.98) to a hyperbolic equation (Equation 7). From the fit, the apparent K_D_^dNTP^ was found to be 4.0±0.3 µM and the maximum concentration of [ED]_A_ ([ED]_A_^max^) was 36±0.5 nM. This dNTP concentration dependence of [ED]_A_ suggests that bond formation is reversible (Figure 2, step 3) and is in equilibrium with ground state dNTP binding (Figure 2, step 2). As a result, [ED]_A_ increases when increasing concentrations of dTTP drive the equilibrium towards product formation. This observation suggests that there is a slow step after catalysis but prior to PPi release that allows chemistry to be reversible.

**Figure 9:**
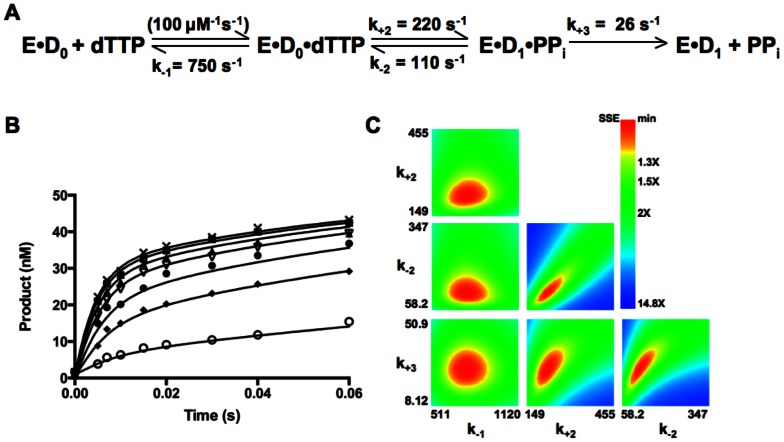
Simulation of kinetic pathway of Sau-PolC- ΔNΔExo. (**A**) The three-step reaction mechanism used for the simulation. Values obtained for the different rate constants are shown alongside the appropriate step. Rate of dNTP association to the E⋅D_0_ binary complex was assumed to be diffusion limited and accordingly the second order rate constant for this step was fixed at 100 µM^−1^s^−1^. (**B**) Simulated curves generated for the representative dataset shown in Figure 8A superimposed on the raw data (concentrations of dTTP shown are ○ 1.17 µM, ♦ 4.69 µM, • 9.4 µM, ▽ 18.75 µM, ▴ 30.14 µM, ▪ 50 µM and ×75 µM). (**C**) 3-D confidence contours for the various rate constants determined from the simulation. For each case the search was carried out up to a sum of squares error (SSE) that is 2-fold higher than the minimum SSE. The upper and lower limits of each parameter were determined using an SSE threshold of 1.2.

We therefore turned to using KinTek Explorer to calculate the kinetic parameters for the forward and reverse steps of chemistry accurately by numerical integration, and also to define a rate constant for the slow step immediately after chemistry. The enzymatic pathway of *Sau*-PolC-ΔNΔExo was simulated through a three-step mechanism: (1) dNTP binding to the polymerase ⋅ DNA binary complex, (2) dNTP incorporation, and (3) PPi release (Figure 9A). We have used this model for simplicity because, for chemistry to be reversible, PPi must be positioned at the active site to cause pyrophosphorolysis. Although PPi release is modeled as a simple binding interaction, it is likely that this step in the pathway also involves a conformational change of the polymerase-DNA complex. Thus, it is important to keep in mind that the kinetic parameters defined for PPi release may actually describe more than one elementary step that occurs immediately after chemistry. Since the ternary complex is stable, as shown in Figure 7A, we assumed that DNA would not dissociate from the enzyme when either dNTP or PPi were bound. For the simulation, the rate of dNTP association was considered to be limited by diffusion and accordingly the second order association rate constant was fixed at 100 µM^−1^s^−1^. Also, PPi release was assumed to be irreversible, since the concentration of PPi in solution during the reaction period would be negligible. The experimentally determined K_D_^DNA^ and DNA release rate (k_off_) from the binary complex were used to determine the concentration of the preformed *Sau*-PolC-ΔNΔExo ⋅ p/t DNA complex and the second order association rate constant for the formation of the binary complex. The simulated curves were generated through iterative steps that used kinetic parameters obtained from nonlinear regression as initial values.

Simulated curves were superimposed on the representative time course data shown in Figure 8A (Figure 9B). Through numerical integration, the rate of chemistry was determined to be ∼220 s^−1^ for the forward reaction and ∼110 s^−1^ for the reverse reaction, yielding a net maximum rate (k_pol_) of ∼330 s^−1^. Also, the rate of pyrophosphate release following chemistry was determined to be 26 s^−1^ and the K_D_^dNTP^ was 7.5 µM. From 3-D confidence contour analysis, all the calculated parameters appeared to be well constrained by the data (Figure 9C). From the simulation, the calculated rate of PPi release is much lower than the calculated rate of catalysis and is very close to the k_cat_ of *Sau*-PolC-ΔNΔExo, suggesting that PPi release (or a conformational change required for PPi release) may govern the steady-state rate.

## Discussion

**Figure 10:**
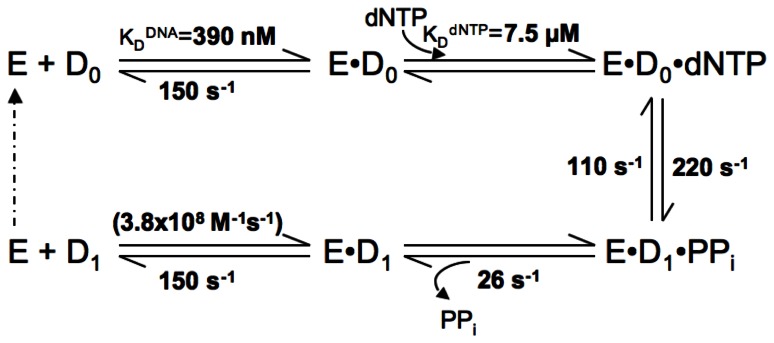
Minimal enzymatic pathway for Sau-PolC- ΔNΔExo. The kinetic parameters determined are shown alongside the corresponding steps of the pathway. K_D_^dNTP^, forward and reverse rates of chemistry and rate of PPi release were derived from simulation of the reaction pathway. The rate of enzyme-DNA association (k_on_) was calculated from the K_D_^DNA^ and k_off_ using the relation that K_D_^DNA^ = k_off_/k_on_. It should be noted that although nucleotide binding and PPi release are each shown as single steps, they may in fact be comprised of more than one elementary step, such as a conformational change in the polymerase accompanying substrate binding and product release.

We have determined the minimal kinetic pathway (Figure 10) for *Sau*-PolC-ΔNΔExo and defined parameters for individual steps within the pathway using both steady-state and pre-steady-state kinetic approaches. The kinetic steps fit the same pathway used by other polymerases, but PolC exhibits several distinguishing features (Table 1). To our knowledge, this is the first comprehensive kinetic study of the catalytic subunit of the bacterial replisome. As described below, our findings provide deeper insight into several activities of the bacterial C-family polymerases that have been previously observed.

Our data show that *Sau*-PolC-ΔNΔExo is a fast enzyme, with a maximum nucleotide incorporation rate k_pol_ of 330 s^−1^, but also binds DNA weakly, with a K_D_^DNA^ of 390 nM, and dissociates rapidly from DNA, with a k_off_ of 150 s^−1^ when dNTP is not bound. These parameters indicate that *Sau*-PolC-ΔNΔExo would have a very low processivity, which is consistent with previous reports about C-family replicative polymerases being non-processive in the absence of accessory protein factors like the β-clamp [12], [35]. Furthermore, weak DNA binding is important for rapid, high fidelity DNA synthesis by bacterial replicative polymerases, as evidenced by an *E. coli* DnaE mutant, *dnaE173*, which has a single amino acid substitution of E612K [36]. The mutant enzyme binds DNA more tightly and simultaneously becomes more processive (even in the absence of clamp), but also shows a reduced rate of DNA elongation and is more error-prone [36]. The data we have obtained for *Sau*-PolC-ΔNΔExo are also consistent with the little kinetic data previously available for full-length C-family polymerases [37]–[39].

The most surprising result from these studies is that nucleotide binding is in equilibrium with the chemical step of the phosphoryl transfer reaction. For this to be the case, the step immediately after chemistry must be slow enough so that there is a build up of the enzyme-DNA-PPi complex resulting from chemistry, thus driving the reverse of nucleotide addition (pyrophosphorolysis). We hypothesize that PPi release after catalysis, or a conformational change that precedes PPi release, might act as a rate-limiting step in the catalytic cycle, allowing the polymerase to retain a conformation favorable to reversal of bond formation. From simulating the reaction mechanism, the rate of this slow step was calculated to be 26 s^−1^ and is likely to be the main determinant of k_cat_ (17 s^−1^).

Equilibrium between chemistry and ground state dNTP binding has not, to our knowledge, been observed previously for standard dNTPs, but has been reported for mitochondrial DNA polymerase gamma incorporating the damaged nucleotide 8-oxo-dGTP and the nucleotide analog AZT-TP [40]–[42]. Since this was not observed with standard nucleotides, it was proposed to be a mechanism for removing non-natural nucleotides. If the slow step after chemistry plays the same role for PolC, it could provide a rudimentary proofreading function.

Previous studies show that the sliding β-clamp processivity factor increases not only the processivity of the C-family polymerases but also the intrinsic rate of nucleotide incorporation. *E. coli* pol III core, for example, synthesizes DNA at a rate of 20 nt/sec, but the rate increases to at least 500 nt/sec in the presence of clamp [43], [44]. Similarly, the intrinsic nucleotide incorporation rate of *S. aureus* PolC on primed circular single-stranded DNA was estimated to be ∼80 nt/sec, and this increased to ∼480 nt/sec in the presence of the sliding clamp [12]. Association with processivity factor, however, does not always stimulate the rate of nucleotide incorporation in this way. The processivity of *E. coli* pol II increases from 5 to ∼1600 nucleotides in the presence of β-clamp, but nucleotides are incorporated at a rate of ∼30 nt/sec in both cases [45]. These observations led to the proposal that β-clamp not only increases the processivity of the C-family replicative polymerases, but that it also increases the rate of a limiting step in the catalytic cycle [12].

Our kinetic data suggest a mechanism by which β-clamp may increase the rate of nucleotide incorporation: by increasing the rate of the slow step immediately after chemistry. β-clamp increases processivity by encircling the DNA duplex and eliminating DNA dissociation as a step in the reaction pathway. However, since *Sau*-PolC-ΔNΔExo has a slow kinetic step (26 s^−1^) between catalysis and DNA dissociation, nucleotide incorporation would not be stimulated if this was the only step in the reaction pathway that was changed. We hypothesize that β-clamp binding to PolC would speed up the rate of the slow step after chemistry, perhaps by stimulating PPi release. If the step immediately after chemistry was no longer rate limiting, we would expect the rate of processive DNA synthesis to increase to the rate of chemistry (330 s^−1^).

Overall, the results presented here establish the kinetic foundation for future structure-function studies of the C-family polymerases by allowing a quantitative comparison of enzyme activities. Structural studies indicate that DnaE and PolC are members of the β-nucleotidyl transferase superfamily, making the bacterial replicative polymerases more closely related to eukaryotic repair polymerases (such as pol β from the X-family) than to eukaryotic or archaeal replicative polymerases [16]–[19]. Some questions that can now be addressed include: How do the C-family polymerases incorporate nucleotides more than 100-fold faster than the X-family polymerases? What contributes to the substantial differences in substrate binding between the two polymerase families? How do the replicative C-family polymerases achieve such a high fidelity of DNA synthesis? PolC has been identified as a novel drug target for antibiotics against Gram-positive bacteria [46], [47]. Beyond increasing our fundamental understanding of bacterial DNA replication, addressing these questions may help in identifying novel features of PolC that could help in developing new antibiotics against Gram-positive pathogens.

## References

[1] Filee J, Forterre P, Sen-Lin T, Laurent J (2002) Evolution of DNA polymerase families: evidences for multiple gene exchange between cellular and viral proteins. J Mol Evol 54: 763–773. 12029358 10.1007/s00239-001-0078-x

[2] Ito J, Braithwaite DK (1991) Compilation and alignment of DNA polymerase sequences. Nucleic Acids Res 19: 4045–4057. 1870963 10.1093/nar/19.15.4045 PMC328540

[3] Huang YP, Ito J (1999) DNA polymerase C of the thermophilic bacterium Thermus aquaticus: classification and phylogenetic analysis of the family C DNA polymerases. J Mol Evol 48: 756–769. 10229580 10.1007/pl00006520

[4] Breier AM, Weier H-UG, Cozzarelli NR (2005) Independence of replisomes in *Escherichia coli* chromosomal replication. Proc Natl Acad Sci U S A 102: 3942–3947. 15738384 10.1073/pnas.0500812102 PMC552787

[5] Bruck I, Georgescu RE, O’Donnell M (2005) Conserved interactions in the *Staphylococcus aureus* DNA PolC chromosome replication machine. J Biol Chem 280: 18152–18162. 15647255 10.1074/jbc.M413595200

[6] Bruck I, O’Donnell M (2000) The DNA replication machine of a gram-positive organism. J Biol Chem 275: 28971–28983. 10878011 10.1074/jbc.M003565200

[7] Bruck I, Yuzhakov A, Yurieva O, Jeruzalmi D, Skangalis M, et al (2002) Analysis of a multicomponent thermostable DNA polymerase III replicase from an extreme thermophile. J Biol Chem 277: 17334–17348. 11859073 10.1074/jbc.M110198200

[8] Bullard JM, Pritchard AE, Song M-S, Glover BP, Wieczorek A, et al (2002) A three-domain structure for the delta subunit of the DNA Polymerase III holoenzyme delta domain III binds delta-prime and assembles into the DnaX complex. J Biol Chem 277: 13246–13256. 11809766 10.1074/jbc.M108708200

[9] Dohrmann PR, Manhart CM, Downey CD, McHenry CS (2011) The rate of polymerase release upon filling the gap between Okazaki fragments is inadequate to support cycling during lagging strand synthesis. J Mol Biol 414: 15–27. 21986197 10.1016/j.jmb.2011.09.039 PMC3236602

[10] Downey CD, Crooke E, McHenry CS (2011) Polymerase chaperoning and multiple ATPase sites enable the *E. coli* DNA polymerase III holoenzyme to rapidly form initiation complexes. J Mol Biol 412: 340–353. 21820444 10.1016/j.jmb.2011.07.051 PMC3197712

[11] Georgescu RE, Kurth I, O’Donnell ME (2012) Single-molecule studies reveal the function of a third polymerase in the replisome. Nat Struct Mol Biol 19: 113–116. 10.1038/nsmb.2179 PMC3721970 22157955

[12] Klemperer N, Zhang D, Skangalis M, O’Donnell M (2000) Cross-utilization of the beta sliding clamp by replicative polymerases of evolutionary divergent organisms. J Biol Chem 275: 26136–26143. 10851235 10.1074/jbc.M002566200

[13] McInerney P, Johnson A, Katz F, O’Donnell M (2007) Characterization of a triple DNA polymerase replisome. Mol Cell 27: 527–538. 17707226 10.1016/j.molcel.2007.06.019

[14] Sanders GM, Dallmann HG, McHenry CS (2010) Reconstitution of the *B. subtilis* replisome with 13 proteins including two distinct replicases. Mol Cell 37: 273–281. 20122408 10.1016/j.molcel.2009.12.025

[15] Yao NY, Georgescu RE, Finkelstein J, O’Donnell ME (2009) Single-molecule analysis reveals that the lagging strand increases replisome processivity but slows replication fork progression. Proc Natl Acad Sci U S A 106: 13236–13241. 19666586 10.1073/pnas.0906157106 PMC2726342

[16] Bailey S, Wing RA, Steitz TA (2006) The structure of *T. aquaticus* DNA polymerase III is distinct from eukaryotic replicative DNA polymerases. Cell 126: 893–904. 16959569 10.1016/j.cell.2006.07.027

[17] Evans RJ, Davies DR, Bullard JM, Christensen J, Green LS, et al (2008) Structure of PolC reveals unique DNA binding and fidelity determinants. Proc Natl Acad Sci U S A 105: 20695–20700. 19106298 10.1073/pnas.0809989106 PMC2634937

[18] Lamers MH, Georgescu RE, Lee SG, O’Donnell M, Kuriyan J (2006) Crystal structure of the catalytic alpha subunit of *E. coli* replicative DNA polymerase III. Cell 126: 881–892. 16959568 10.1016/j.cell.2006.07.028

[19] Wing RA, Bailey S, Steitz TA (2008) Insights into the replisome from the structure of a ternary complex of the DNA polymerase III alpha-subunit. J Mol Biol 382: 859–869. 18691598 10.1016/j.jmb.2008.07.058 PMC2614274

[20] Johnson A, O’Donnell M (2005) Cellular DNA replicases: components and dynamics at the replication fork. Annu Rev Biochem 74: 283–315. 15952889 10.1146/annurev.biochem.73.011303.073859

[21] Johnson KA (1993) Conformational coupling in DNA polymerase fidelity. Annu Rev Biochem 62: 685–713. 7688945 10.1146/annurev.bi.62.070193.003345

[22] Johnson KA (2010) The kinetic and chemical mechanism of high-fidelity DNA polymerases. Biochim Biophys Acta 1804: 1041–1048. 20079883 10.1016/j.bbapap.2010.01.006 PMC3047511

[23] Joyce CM (2010) Techniques used to study the DNA polymerase reaction pathway. Biochim Biophys Acta 1804: 1032–1040. 19665596 10.1016/j.bbapap.2009.07.021 PMC2846202

[24] Kuchta RD, Mizrahi V, Benkovic PA, Johnson KA, Benkovic SJ (1987) Kinetic mechanism of DNA polymerase I (Klenow). Biochemistry 26: 8410–8417. 3327522 10.1021/bi00399a057

[25] Patel SS, Wong I, Johnson KA (1991) Pre-steady-state kinetic analysis of processive DNA replication including complete characterization of an exonuclease-deficient mutant. Biochemistry 30: 511–525. 1846298 10.1021/bi00216a029

[26] Doublie S, Tabor S, Long AM, Richardson CC, Ellenberger T (1998) Crystal structure of a bacteriophage T7 DNA replication complex at 2.2 Å resolution. Nature 391: 251–258. 9440688 10.1038/34593

[27] Li Y, Korolev S, Waksman G (1998) Crystal structures of open and closed forms of binary and ternary complexes of the large fragment of *Thermus aquaticus* DNA polymerase I: structural basis for nucleotide incorporation. EMBO J 17: 7514–7525. 9857206 10.1093/emboj/17.24.7514 PMC1171095

[28] Joyce CM, Potapova O, Delucia AM, Huang X, Basu VP, et al (2008) Fingers-closing and other rapid conformational changes in DNA polymerase I (Klenow fragment) and their role in nucleotide selectivity. Biochemistry 47: 6103–6116. 18473481 10.1021/bi7021848

[29] Rothwell PJ, Mitaksov V, Waksman G (2005) Motions of the fingers subdomain of Klentaq1 are fast and not rate limiting: implications for the molecular basis of fidelity in DNA polymerases. Mol Cell 19: 345–355. 16061181 10.1016/j.molcel.2005.06.032

[30] Tsai YC, Johnson KA (2006) A new paradigm for DNA polymerase specificity. Biochemistry 45: 9675–9687. 16893169 10.1021/bi060993z PMC7526746

[31] Kellinger MW, Johnson KA (2010) Nucleotide-dependent conformational change governs specificity and analog discrimination by HIV reverse transcriptase. Proc Natl Acad Sci U S A 107: 7734–7739. 20385846 10.1073/pnas.0913946107 PMC2867896

[32] Johnson KA, Simpson ZB, Blom T (2009) FitSpace explorer: an algorithm to evaluate multidimensional parameter space in fitting kinetic data. Anal Biochem 387: 30–41. 19168024 10.1016/j.ab.2008.12.025

[33] Johnson KA, Simpson ZB, Blom T (2009) Global kinetic explorer: a new computer program for dynamic simulation and fitting of kinetic data. Anal Biochem 387: 20–29. 19154726 10.1016/j.ab.2008.12.024

[34] Johnson KA (1992) 1 Transient-State Kinetic Analysis of Enzyme Reaction Pathways. In: David SS, editor. The Enzymes: Academic Press. 1–61.

[35] Fay PJ, Johanson KO, McHenry CS, Bambara RA (1981) Size classes of products synthesized processively by DNA polymerase III and DNA polymerase III holoenzyme of *Escherichia coli* . J Biol Chem 256: 976–983. 7005228

[36] Yanagihara F, Yoshida S, Sugaya Y, Maki H (2007) The dnaE173 mutator mutation confers on the alpha subunit of *Escherichia coli* DNA polymerase III a capacity for highly processive DNA synthesis and stable binding to primer/template DNA. Genes Genet Syst 82: 273–280. 17895578 10.1266/ggs.82.273

[37] Burrows JA, Goward CR (1992) Purification and properties of DNA polymerase from *Bacillus caldotenax* . Biochem J 287 (Pt 3): 971–977. 10.1042/bj2870971 PMC1133102 1445254

[38] Kim DR, Pritchard AE, McHenry CS (1997) Localization of the active site of the alpha subunit of the *Escherichia coli* DNA polymerase III holoenzyme. J Bacteriol 179: 6721–6728. 9352922 10.1128/jb.179.21.6721-6728.1997 PMC179601

[39] Shapiro A, Rivin O, Gao N, Hajec L (2005) A homogeneous, high-throughput fluorescence resonance energy transfer-based DNA polymerase assay. Anal Biochem 347: 254–261. 16266678 10.1016/j.ab.2005.09.023

[40] Hanes JW, Johnson KA (2007) A novel mechanism of selectivity against AZT by the human mitochondrial DNA polymerase. Nucleic Acids Res 35: 6973–6983. 17940100 10.1093/nar/gkm695 PMC2175305

[41] Hanes JW, Thal DM, Johnson KA (2006) Incorporation and replication of 8-oxo-deoxyguanosine by the human mitochondrial DNA polymerase. J Biol Chem 281: 36241–36248. 17005553 10.1074/jbc.M607965200

[42] Johnson AA, Ray AS, Hanes J, Suo Z, Colacino JM, et al (2001) Toxicity of antiviral nucleoside analogs and the human mitochondrial DNA polymerase. J Biol Chem 276: 40847–40857. 11526116 10.1074/jbc.M106743200

[43] Studwell PS, O’Donnell M (1990) Processive replication is contingent on the exonuclease subunit of DNA polymerase III holoenzyme. J Biol Chem 265: 1171–1178. 2153103

[44] Maki H, Kornberg A (1985) The polymerase subunit of DNA polymerase III of *Escherichia coli*. II. Purification of the alpha subunit, devoid of nuclease activities. J Biol Chem 260: 12987–12992. 2997151

[45] Bonner CA, Stukenberg PT, Rajagopalan M, Eritja R, O’Donnell M, et al (1992) Processive DNA synthesis by DNA polymerase II mediated by DNA polymerase III accessory proteins. J Biol Chem 267: 11431–11438. 1534562

[46] Standish AJ, Salim AA, Capon RJ, Morona R (2013) Dual inhibition of DNA polymerase PolC and protein tyrosine phosphatase CpsB uncovers a novel antibiotic target. Biochem Biophys Res Commun 430: 167–172. 23194664 10.1016/j.bbrc.2012.11.049

[47] Yang F, Dicker IB, Kurilla MG, Pompliano DL (2002) PolC-type polymerase III of *Streptococcus pyogenes* and its use in screening for chemical inhibitors. Anal Biochem 304: 110–116. 11969194 10.1006/abio.2001.5591

[48] Lowe LG, Guengerich FP (1996) Steady-state and pre-steady-state kinetic analysis of dNTP insertion opposite 8-oxo-7,8-dihydroguanine by *Escherichia coli* polymerases I exo- and II exo. Biochemistry 35: 9840–9849. 8703958 10.1021/bi960485x

[49] Einolf HJ, Guengerich FP (2001) Fidelity of nucleotide insertion at 8-oxo-7,8-dihydroguanine by mammalian DNA polymerase delta. Steady-state and pre-steady-state kinetic analysis. J Biol Chem 276: 3764–3771. 11110788 10.1074/jbc.M006696200

[50] Dieckman LM, Johnson RE, Prakash S, Washington MT (2010) Pre-steady state kinetic studies of the fidelity of nucleotide incorporation by yeast DNA polymerase delta. Biochemistry 49: 7344–7350. 20666462 10.1021/bi100556m PMC2941984

[51] Graves SW, Johnson AA, Johnson KA (1998) Expression, purification, and initial kinetic characterization of the large subunit of the human mitochondrial DNA polymerase. Biochemistry 37: 6050–6058. 9558343 10.1021/bi972685u

[52] Johnson AA, Tsai Y, Graves SW, Johnson KA (2000) Human mitochondrial DNA polymerase holoenzyme: reconstitution and characterization. Biochemistry 39: 1702–1708. 10677218 10.1021/bi992104w

[53] Werneburg BG, Ahn J, Zhong X, Hondal RJ, Kraynov VS, et al (1996) DNA polymerase beta: pre-steady-state kinetic analysis and roles of arginine-283 in catalysis and fidelity. Biochemistry 35: 7041–7050. 8679529 10.1021/bi9527202

[54] Bertram JG, Bloom LB, O’Donnell M, Goodman MF (2004) Increased dNTP binding affinity reveals a nonprocessive role for *Escherichia coli* beta clamp with DNA polymerase IV. J Biol Chem 279: 33047–33050. 15210708 10.1074/jbc.C400265200

